# Dietary Uptake of *Wedelia chinensis* Extract Attenuates Dextran Sulfate Sodium-Induced Colitis in Mice

**DOI:** 10.1371/journal.pone.0064152

**Published:** 2013-05-29

**Authors:** Yuh-Ting Huang, Chih-Chun Wen, Yung-Hsiang Chen, Wen-Ching Huang, Li-Ting Huang, Wen-Ching Lin, Palanisamy Arulselvan, Jiunn-Wang Liao, Shu-Hui Lin, Pei-Wen Hsiao, Sheng-Chu Kuo, Ning-Sun Yang

**Affiliations:** 1 Agricultural Biotechnology Research Center, Academia Sinica, Taipei, Taiwan; 2 Graduate Institute of Pharmaceutical Chemistry, China Medical University, Taichung, Taiwan; 3 Graduate Institute of Veterinary Pathology, National Chung Hsing University, Taichung, Taiwan; 4 One Power Bio Technology Co., Ltd., Taipei, Taiwan; Charité, Campus Benjamin Franklin, Germany

## Abstract

**Scope:**

Traditional medicinal herbs are increasingly used as alternative therapies in patients with inflammatory diseases. Here we evaluated the effect of *Wedelia chinensis*, a medicinal herb commonly used in Asia, on the prevention of dextran sulfate sodium (DSS)-induced acute colitis in mice. General safety and the effect of different extraction methods on the bioactivity of *W. chinensis* were also explored.

**Methods and Results:**

C57BL/6 mice were administrated hot water extract of fresh *W. chinensis* (WCHF) orally for one week followed by drinking water containing 2% DSS for nine days. WCHF significantly attenuated the symptoms of colitis including diarrhea, rectal bleeding and loss of body weight; it also reduced the shortening of colon length and histopathological damage caused by colonic inflammation. Among four *W. chinensis* extracts prepared using different extraction techniques, WCHF showed the highest anti-colitis efficacy. Analyses of specific T-cell regulatory cytokines (TNF-α, IL-4, IFN-γ, IL-17, TGF-β, IL-12) revealed that WCHF treatment can suppress the Th1 and Th17, but not Th2, responses in colon tissues and dendritic cells of DSS-induced colitis mice. A 28-day subacute toxicity study showed that daily oral administration of WCHF (100, 500, 1000 mg/kg body weight) was not toxic to mice.

**Conclusion:**

Together, our findings suggest that specific extracts of *W. chinensis* have nutritional potential for future development into nutraceuticals or dietary supplements for treatment of inflammatory bowel disease.

## Introduction

Inflammatory bowel disease (IBD), including Crohn’s disease (CD) and ulcerative colitis (UC), represents a group of chronic relapsing inflammatory disorders of the gastrointestinal tract that affect millions of people worldwide. Both CD and UC are characterized by mucosal inflammation, crypt destruction, infiltration of leukocytes and features such as diarrhea, rectal bleeding, abdominal pain and weight loss [1]–[3]. Patients with IBD do not only suffer from the clinical symptoms, but are also at a high risk of developing colorectal cancer [4], [5]. Incidence of IBD, especially UC, remains relatively constant in regions like Northern Europe and North America; however, it is increasing in the areas where incidence was previously low, such as Southern Europe and Asia [6]. Evidence from epidemiological and pathogenesis studies has shown that IBD is associated with a complex interaction of environmental triggers (such as diet and smoking), familial and genetic factors, immunoregulatory defects and microbial exposure [1], [7], which result in an inappropriate and ongoing activation of the mucosal immune system.

Although the exact pathogenesis of IBD is not yet clear, infiltration of neutrophils, activation of macrophages and unregulated production of pro-inflammatory molecules in inflamed colon epithelial tissues are thought to be crucial factors. Conventional drugs used for treatment of IBD are mostly anti-inflammatory or immunomodulatory agents, including corticosteroids, and 5-amino salicylic acid (5-ASA) and its derivatives (such as sulfasalazine). 5-ASA-based drugs are prescribed most frequently for IBD [8], but they can induce side effects including nausea, headache, heartburn and anemia. Long-term high-doses of corticosteroids can also cause serious side effects, notably Cushing’s syndrome [9]. Therefore, novel therapeutics or preventive treatments that are nontoxic and yet can effectively decrease mucosal inflammation with few or no side effects are highly desirable.

In recent years, natural health-care products derived from medicinal plants or herbs have been developed as alternative or complementary treatments for many common disorders. Two recent surveys reported that among IBD patients, the most frequently used types of complementary and alternative medicine (CAM) are herbal remedies [10], [11]. *Wedelia chinensis* (a Compositae) is a key traditional medicinal herb that is widely used in many Asia countries, and often serves as a major component of folk herbal teas. In Taiwan and Southeast Asia, *W. chinensis* is considered to have various therapeutic properties such as cough-relieving, antipyretic, detoxication, antiphlogistic [12], and to confer a hepato-protective effect, as shown in mice with acute hepatitis induced by hepatotoxins [13]. Compounds found in *W. chinensis* have been recently reported to attenuate androgen receptor activity and orthotropic growth of prostate cancer in nude mice via the inhibition of androgen receptor signaling pathway [14]. We therefore hypothesized that specific *W. chinensis* plant extracts may confer anti-inflammatory activity against IBD.

In the present study, the effects of orally fed plant extracts of *W. chinensis*, prepared by different extraction methods were analyzed in a dextran sulfate sodium (DSS)-induced colitis mouse model. Optimum daily dosage, general safety, metabolite profiles and candidate active components of this herbal plant were evaluated and identified. Possible applications of the results in future development of nutritional and medicinal food supplements for colon health care are discussed.

## Materials and Methods

### Preparation of *W. chinensis* Extracts

*W. chinensis* was routinely obtained from a reputable Chinese medicinal herb store/farmer in Taipei City, Taiwan, and the experimental plant materials were validated macroscopically by specific morphology, anatomy, phytochemistry and genome sequence features as previously reported [15], [16], and from our own studies (Lin et al., submitted for publication) [17]. Dried *W. chinensis* was prepared by air drying cleaned fresh plants in the shade for two weeks. To prepare the hot water extracts of fresh or dried *W. chinensis* plants (WCHF and WCHD, respectively), test plant materials were weighed, and decocted in appropriate volume (100 g fresh or dried WC in 1 L water) of boiling water, and continuously boiled until the volume was reduced to one-fourth of the original. Boiling water extract was then filtered through filter papers (No. 2, Toyo Roshi Kaisha, Tokyo, Japan) using a suction pump, and concentrated using a rotary evaporator. Ethanol extracts were obtained by immersing the whole fresh *W. chinensis* plants in 100% or 50% EtOH (WC100 or WC50, respectively) for two weeks, and filtering and concentrating as described above. All *W. chinensis* extracts were then freeze-dried and stored at 4°C before use.

### Mice

**Figure 1:**
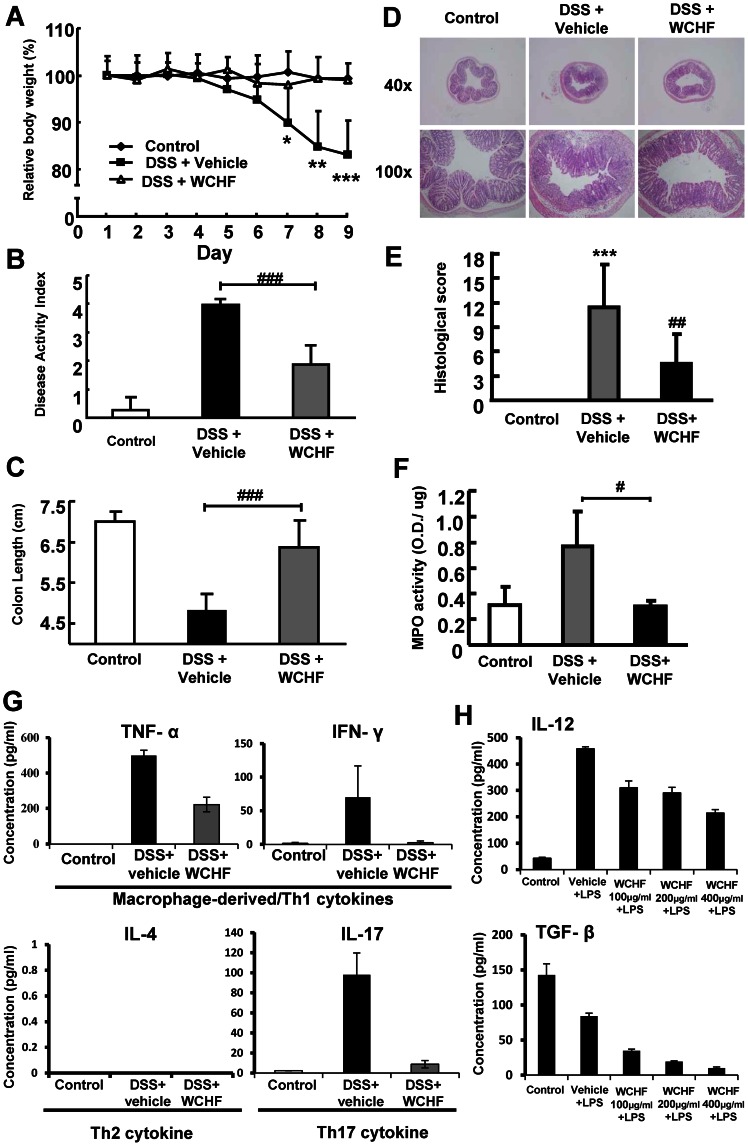
Effect of dietary WCHF in mice with DSS-induced acute colitis. Colitis was induced by providing 2% DSS in drinking water for 9 days. Mice were simultaneously orally administered with vehicle (1% Tween 80) or WCHF (50 mg/kg body weight) respectively for 7 days, followed by DSS treatment for 9 days (test mouse were administered continuously with test sample during the DSS treatment). Changes of (A) relative body weight, (B) disease activity index, (C) colon length, (D) histological section, (E) histological score and (F) myeloperoxidase activity are shown. Data are expressed as mean ± SD. (n = 5–6). (G) Effect of hot water extract of fresh *W. chinensis* (WCHF) on expression of specific cytokines and T cells response in WCHF-treated and DSS-induced colitis mice. (H) Effect of WCHF extract (100 µg/ml, 200 µg/ml and 400 µg/ml) on *ex-vivo* expression of IL-12 and TGF-β from mouse DCs in culture. Representative H&E-stained middle tissue sections of colons under 40× and 100× magnification. *P<0.05, **P<0.005, ***P<0.001, significant difference compared with the control group. ^#^ *P*<0.05, ^##^ *P*<0.005, ^###^ *P*<0.001, significant difference compared with the DSS group.

**Figure 2:**
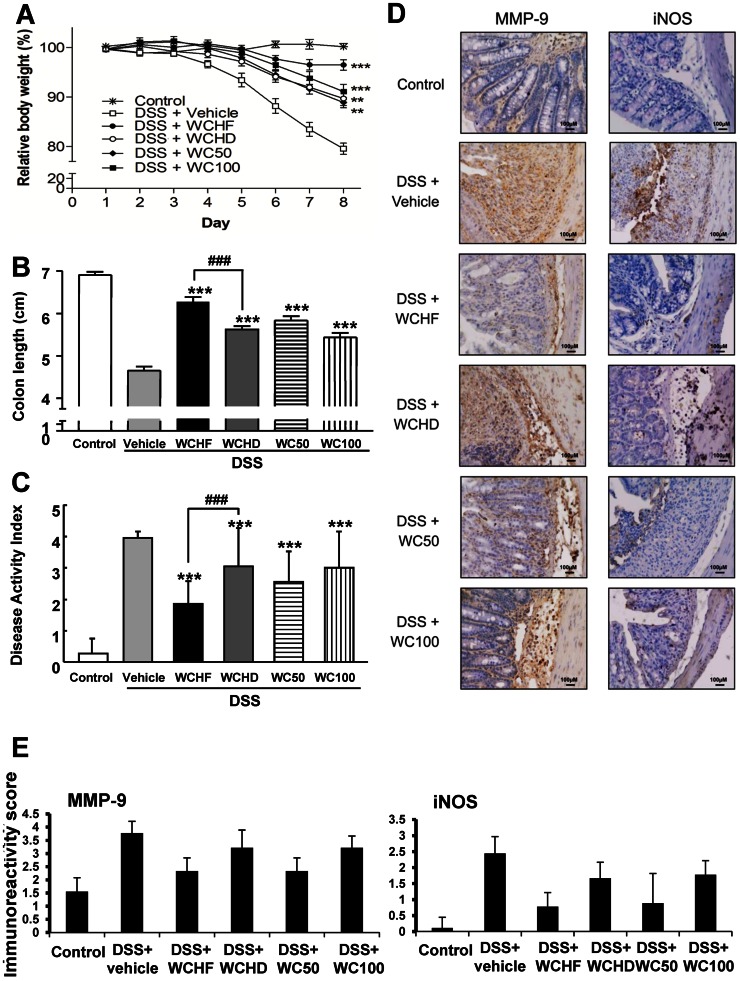
Effects of different *W. chinensis* extracts on mice with DSS-induced acute colitis. *W. chinensis* extracts were prepared by different extraction methods as described in Materials and Methods. Mice were simultaneously orally administered with vehicle (1% Tween 80) or different *W. chinensis* extracts (50 mg/kg body weight) respectively for 7 days, followed by DSS treatment for 9 days (test mouse were administered continuously with test samples during the DSS treatment). Changes of (A) relative body weight, (B) colon length, and (C) disease activity index are shown. Data are expressed as mean ± SD. (n = 5–6). (D) Determination of MMP-9 and iNOS by immunohistochemical analysis. Positive staining was seen as brown for both MMP-9 and iNOS. Representative colon tissue sections are shown at 200× magnification. (E) MMP-9 and iNOS immunoreactivity (represented by the intensity of brown staining) was scored. WCHF: hot water extract of fresh *W. chinensis*; WCHD: hot water extract of dried *W. chinensis*; WC50 and WC100∶50% and 100% ethanol extracts of *W. chinensis*, respectively **P<0.005, ***P<0.001, significant difference compared with the DSS group.

Seven- to eight-week-old female C57BL/6 and ICR mice were purchased from the National Laboratory Animal Center (Taipei, Taiwan) and maintained on a 12-h light/dark cycle in constant temperature and humidity. Mice were given food and water *ad libitum* until they reached the desired weight for experiments. All procedures were approved by the Institutional Animal Care and Use Committee (IACUC) of Academia Sinica (Protocol ID: 10-12-098). In this study, two experimental groups (see Fig. 1A and Fig. 2A), some test mice lost 21% to 28% of their body weight on Day 6∼8 were allowed to live for 1∼2 extra days, and were sacrificed on the end of the experiment (Day 8 or Day 9). This measure has obtained the appropriate and careful guidance from the institutional IACUC and animal room specialist, and we consider these cases as extremely rare but necessary and humane arrangement.

### Induction of Acute Colitis

After acclimatization, weight-matched female C57BL/6 mice (20–25 g) were randomized into control (untreated), colitis-control, and treatment groups. All mice received normal drinking water from day −7 to 0. To induce acute colitis, all mice, except for the control group, were provided with drinking water containing 2% (w/v) DSS (molecular weight: 36,000–50,000 Da, MP Biochemicals, Solon, Ohio) from day 0 to 8–9 as previously described [18]. DSS solution was changed every two days. Throughout the whole experimental period (−7th to 8th∼9th day post DSS treatment), mice in the control and DSS groups were daily administered orally with vehicle control solution (1% Tween 80, Sigma-Aldrich, St. Louis, MO). Treatment groups were fed daily by oral gavage with different preparations of *W. chinensis* extracts dissolved in vehicle solution at a dose of 50 mg/kg body weight. To assess the extent of colitis, body weight, stool consistency, and fecal blood levels were monitored daily, and the disease activity index (DAI) of colitis was calculated according to the modified criteria outlined in Cooper et al. [19]. At the end of each experiment, all test mice were weighed and sacrificed by cervical dislocation and the large intestines were excised; the colon length was measured and the cecum was removed. The remainder was washed with 0.9% saline solution and fixed in 10% neutral-buffered formalin.

### Colon Organ Culture

Colon organ cultures were generated and modified as described previously by Wirtz et al. [18]. At the end of acute colitis experiment, freshly excised colon tissues from control and test mice were flushed with sterilized PBS to remove soiling. The colon segment was opened up by a longitudinal incision and the exposed colon tissues minced into small pieces (1 to 2 mm in length). These minced colon tissues were cultured in “organoid” primary culture mode using RPMI 1640-based culture medium supplemented with 10% FBS [18]. At 24 hours post incubation, the test culture media were collected and specific cytokines measured by ELISA.

### Mouse Bone Marrow-derived Dendritic Cells (BMDCs)

Mouse bone marrow-derived DCs were generated and grown in cell cultures as previously described [20]. Briefly, bone marrow tissues were collected from femurs and tibiae of C57BL/6 mice. Erythrocytes were removed from the bone marrow cells by ACK lysis buffer and plated in DC culture medium (RPMI 1640 medium supplemented with 20 ng/ml GM-CSF, 10% fetal bovine serum, 50 µM 2-mercaptoethanol, 100 µM non-essential amino acids and 100 unit/ml penicillin and 100 µg/ml streptomycin) in a humidified 5% CO2 incubator at 37°C. On day 2, two-thirds of original medium was replaced by 30 ml fresh medium supplemented with 20 ng/ml GM-CSF. On day 5, the floating cells were gently removed and the fresh medium was replenished with 20 ng/ml GM-CSF and 20 ng/ml IL-4. On day 7, non-adherent DCs in culture were harvested. The DCs generated in this manner were immature DCs and displayed typical morphologic features of DCs.

### Histopathological Evaluation of Colitis

Histological examination of colons was performed by a veterinary pathologist, (Dr. Jiunn-Wang Liao, Graduate Institute of Veterinary Pathobiology, National Chung Hsing University, Taiwan) and histological score of the middle segment of the colon was determined according to the scoring system described in Cooper et al. [19]. Briefly, formalin-fixed colon tissues were divided into three equal segments (proximal, middle, and distal), embedded in paraffin, sectioned (2 µm) and stained with hematoxylin and eosin (H&E). Histological examination was performed by an independent investigator blinded to the type of treatment. Lesions of DSS-induced colitis were graded as crypt loss, crypt regeneration, edema in the submucosa, inflammation and ulcer (with fibroblast cell infiltration). Degree of lesions was graded from one to five depending on the severity according to the methods described in Shackelford et al. [21]. The histological score was calculated as outlined in Table S1 (Table S1 in File S1), with a maximum score of 25.

### Myeloperoxidase Activity

Myeloperoxidase (MPO) activity, as a biochemical marker for neutrophil accumulation, was determined according to the protocol reported by Krawisz et al. [22] slightly modified. The middle segment of the colon tissue frozen in liquid nitrogen was ground and homogenized in 50 mM potassium phosphate buffer (KH_2_PO_4_/K_2_HPO_4_, pH 6.0) containing 0.5% (w/v) hexadecyltrimethyl ammonium bromide (HETAB). The homogenate was centrifuged at 4°C, 13000 rpm for 20 minutes to collect the supernatant. MPO activity in the supernatant was determined by mixing 100 µl of the supernatant with 50 µl of ready-to-use tetramethylbenzidine (TMB) substrate (Clinical Science Products, MA) in a 96-well plate, reacted for 2 minutes and stopped by adding 50 µl of 2 M sulfuric acid. The optical density (O.D.) was immediately measured using a PowerWave™ XS Microplate spectrophotometer (Bio-Tek, Winooski, VT) at 450 nm. Protein concentration of the supernatant was estimated by Coomassie Protein Assay Reagent (Thermo, Rockford, IL). MPO activity was expressed as O.D./µg protein.

### ELISA Analysis of Inflammatory Cytokines

The levels of cytokine in culture media were measured using an enzyme linked immunosorbent assay (ELISA) kit (R&D systems, Minneapolis, MN) according to the manufacturer’s protocol. All assays were performed in triplicate.

### Immunohistochemical Staining

Expression of inflammatory markers matrix metalloproteinase-9 (MMP-9) and inducible nitric oxide synthase (iNOS) in test tissue were evaluated using immunohistochemical staining as described previously [23]. In brief, colon tissues were cut into 4 µm sections and deparaffinized. After treatment with citrate buffer (pH 6.0), tissue slides were treated with blocking buffer for 1 hour and incubated overnight with primary antibodies against MMP-9 (Cell Signaling Technology, Beverly, MA) and iNOS (Abcam, Cambridge, UK) at 4°C. Slides were washed in PBS and then incubated with secondary antibodies conjugated with horseradish peroxidase (Santa Cruz Biotechnology Inc., Santa Cruz, CA). After washing with PBS again, color was developed using 3, 3′-diaminobenzidine (BD Biosciences, San Jose, CA) as a chromogen, followed by counterstaining with H&E. Photomicrographs were taken using an Olympus DP-70 camera on an Eclipse E800 microscope (Nikon, Tokyo, Japan). The immunoreactivity score was semi-quantified by using the method of Raina [24]. Immunoreactivity (represented by the intensity of brown staining levels) was scored as 0 (no staining), +1 (very weak), +2 (weak), +3 (moderate) and +4 (strong staining).

### High Performance Liquid Chromatography

All high performance liquid chromatography (HPLC)-grade solvents and chemicals were purchased from J.T. Baker (Phillipsburg, NJ). The freeze-dried hot water extracts of fresh and dried *W. chinensis* (WCHF and WCHD, respectively) were ground into powders and dissolved in dimethyl sulfoxide (DMSO, Tedia, Fairfield, OH) as stocks (20 mg/ml). Aliquots of WCHF or WCHD were diluted into 1 mg/ml in methanol and centrifuged at 13000 rpm for 10 minutes to collect the supernatant. For each extract, 20 µl supernatant was analyzed by HPLC (Agilent 1100 Series System with G131A Quat Pump, Palo Alto, CA) using a Cosmosil C_18_-AR-II column (5 µm particle size, 250×4.6 mm, Nacalai Tesque, Kyoto, Japan) at a stable flow rate of 1 ml/min. Gradient elution was performed with a mobile phase consisting of methanol (solvent B) and 0.05% trifluoroacetic acid (TFA, Alfa Aesar, Ward Hill, MA) in deionized distilled water (solvent A): 0–30 min, 15%–100% solvent B and 30–40 min, 100% solvent B. Detection wavelengths were set at 254, 280, 300 and 350 nm.

### Subacute Oral Toxicity Study

Weight-matched ICR mice were randomly divided into four groups and administered orally with water (control group) or 100, 500, 1000 mg/kg body weight of WCHF (as low, middle, and high-dose groups, respectively) for 28 days. Body weight and food consumption were measured. Clinical signs and mortality were observed twice daily. At the end of 28 days, mice were sacrificed. Organs were observed macroscopically and selected vital organs (heart, liver, spleen, lung and kidney) were weighed and fixed in 10% neutral-buffered formaldehyde solution. Hematological and biochemical analyses of blood and urine were performed by the Taiwan Mouse Clinic. Histological examination was performed by Dr. Jiunn-Wang Liao.

### Statistical Analysis

Results are expressed as mean ± S.D. of a representative experiment performed in triplicate. Statistical analysis was performed using an unpaired, two-tailed Student’s *t*-test.

## Results

### WCHF Ameliorates the Symptoms of DSS-induced Acute Colitis

Effect of the hot water extract of fresh *W. chinensis* plant (WCHF) was evaluated in a DSS-induced acute colitis mouse model. Test mice were treated orally with either vehicle solution or WCHF extract (50 mg/kg body weight) from the beginning of the experiment for 1 week, followed by provision of 2% DSS in drinking water for 9 days. Mice in the DSS group soon developed typical symptoms of clinical colitis, including diarrhea, rectal bleeding and loss of body weight. As shown in Fig. 1A, compared with the control group, the relative body weight (%) of the DSS-treated group decreased significantly on day 7, 8, and 9 (*P*<0.05, *P*<0.005, *P*<0.001, respectively). Mice in the WCHF group showed no difference in body weight from the control group. The disease activity index was found to be significantly lower in the WCHF+DSS-treated mice than that of mice treated with DSS alone (Fig. 1B, *P*<0.001).

Besides the loss of body weight, shortening of colon length is another important symptomatic parameter of DSS-induced colitis [19]. As seen in Fig. 1C, WCHF treatment effectively reduced the shortening of colon length in test mice (*P*<0.001). It also attenuated histopathological manifestation (Fig. 1D). As compared with the typical H&E-stained mid-colon tissue sections taken from mice treated with DSS, histological sections from WCHF-treated mice showed a markedly lower degree of crypt destruction and infiltration of mononuclear cells or other lymphocytes into the mucosal tissue (Fig. 1D). The histological scores were also substantially decreased in the WCHF-treated group (*P*<0.005, Fig. 1E). We subsequently collected colon tissues and analyzed the levels of myeloperoxidase (MPO) activity in test samples. Consistent with the histological scores, MPO activity was greatly increased in the DSS group, but the activity in the WCHF group was effectively reduced to the level of the untreated (control) group (*P*<0.05, Fig. 1F). Together, our results (Fig. 1A–F) show that WCHF treatment at a dose of 50 mg/kg can markedly ameliorate both the clinical symptoms and the tissue damage caused by DSS-induced inflammation and wounding in test mouse colon tissues.

For inflammatory bowel disease (IBD), specific patho-/physio-logical and dysregulated immunogenic responses have been shown to be reflected by specific cytokine profiles at different stages of the IBD process [25]. At the end of specific acute colitis experiments, we tested the effect of hot water extract of fresh *W. chinensis* (WCHF) on expression of specific cytokines and on T cell response in WCHF-treated DSS-induced colitis mice. The minced colon tissues from control and test mice were placed and tested in an “organoid” primary culture mode [18]. At 24 hours post incubation, the organ culture media were collected and specific cytokines measured by ELISA. Fig. 1G shows that WCHF treatment can effectively suppress the DSS-induced increase on expression of macrophage-derived/Th1 **(**TNF-α, IFN-γ**)** and Th17 (IL-17) cytokines, but has no effect on expression of IL-4, the marker of Th2 cytokines. Previous study by Alex et al. [26] has shown that DSS-induced acute colitis can exhibit Th1and Th17-mediated acute inflammation activities (i.e., increase in TNF-α and IL-17 levels). We show here that WCHF treatment results in effective suppression of Th1 and Th17 responses in DSS-induced colitis mice, we hence suggest that WCHF extract can confer anti-colitis activity at least in part via the inhibition of the Th1 and Th17 responses. Furthermore, dendritic cells (DCs) are known to involve in the progression of DSS-induced colitis [27], and IL-12 and TGF-β produced by DCs have been shown to direct the development of Th1 (IL-12) and Th17 (TGF-β) responses [28], [29]. We hence also tested the effect of WCHF extract on expression of specific cytokines from DCs *ex vitro*. Bone marrow-derived DCs were generated as previously described [20], and DCs were pre-treated with WCHF for 2 hours, followed by stimulation with LPS for 24 hours (WCHF was shown first as no-toxic to test DCs, as assayed with trypan blue staining). Culture media from test DCs were then collected for cytokine analyses. Test results showed that WCHF, in a dose-dependent manner, effectively suppressed the secretion of IL-12 and TGF-β in LPS-stimulated DCs (Fig. 1H). These results lead us to suggest that WCHF can inhibit both Th1 and Th17-related cytokine expression in both colon tissues and dendritic cells of test mice, and these bio-activities may play a role in the amelioration of DSS-induced colitis.

### Among Four Tested *W. chinensis* Extracts, WCHF Provides Most Protection Against Colitis

In order to evaluate different extraction methods for optimized preparation of tissue extracts of *W. chinensis* for future application, we investigated four different extraction protocols for preparation of crude plant extracts, and evaluated their effect on anti-colitis activity in mice. Compared with mice treated with DSS only, mice treated with different *W. chinensis* extracts effectively attenuated the effects of DSS on reduction of body weight (Fig. 2A), colon length (Fig. 2B) and the disease activity index (Fig. 2C). Among the four tested extracts (hot water extract of fresh *W. chinensis*, WCHF; hot water extract of dried *W. chinensis*, WCHD; and 100% and 50% ethanol extracts of fresh *W. chinensis*, WC100 and WC50, respectively), WCHF exhibited the highest anti-colitis efficacy. Immunohistochemical staining showed that MMP-9 and iNOS expression in test colon tissues were downregulated by treatment with *W. chinensis* extracts, especially by WCHF (Fig. 2D, 2E). These results are in good agreement with the observations obtained at the organ level (Fig. 2A, 2B, 2C) in this murine colitis model.

### HPLC Analyses of Phytochemicals in *W. chinensis* Plant Extracts

**Figure 3:**
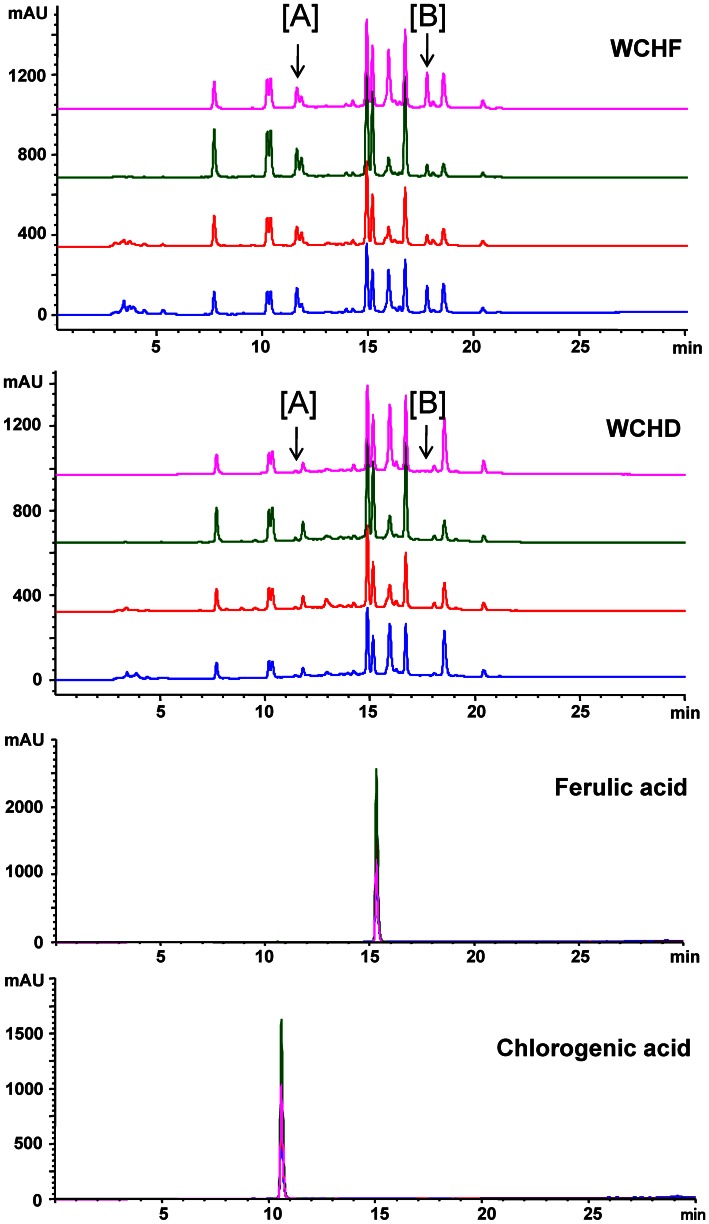
HPLC profiles of the hot water extracts of fresh and dried *W. chinensis* and the candidate chemical markers/index compounds. WCHF, WCHF and the pure single compounds were detected at different wavelengths (blue = 280 nm, red = 254 nm, green = 300 nm, pink = 350 nm). Arrows indicate the specific peaks found in the profile of WCHF that were reduced or virtually absent in that of WCHD.

We used HPLC to analyze the metabolite profile of the hot water extracts of fresh versus dried *W. chinensis*. Two major peaks, detected at retention times of 11.58 minutes (A) and 17.75 minutes (B), present in the WCHF sample were significantly decreased in the metabolite profile of WCHD sample (Fig. 3), suggesting that the presence of these two specific plant metabolite constituents may be associated with the improved anti-colitis activity observed for WCHF.

We additionally compared the HPLC profile of WCHF to several known phytochemicals. Similar retention times indicated that chlorogenic acid and ferulic acid could be the possible metabolites present or enriched in the WCHF preparation, although these two secondary plant metabolites were apparently also present in the WCHD preparation. Previous studies by our own and other laboratories [30]–[32] have shown that ferulic acid and chlorogenic acid can confer specific pharmacological bioactivities. We therefore tested directly the bio-activities of chlorogenic acid and ferulic acid on possible anti-colitis activity. Results from this experiment (data not shown) revealed that, based on measurements of relative body weight, disease activity index and colon length, oral feeding treatment individually with chlorogenic acid or ferulic acid (50 mg/kg body weight) did not result in a significant effect on DSS-induced colitis in test mice. Therefore, the bio-active compound(s) of WCHF (including the unidentified single compounds present in peaks A and B of Fig. 3) that ameliorate the symptoms of DSS-induced acute colitis remains to be unknown to us.

### Daily Oral Administration of WCHF is not Toxic to Test Mice

**Figure 4:**
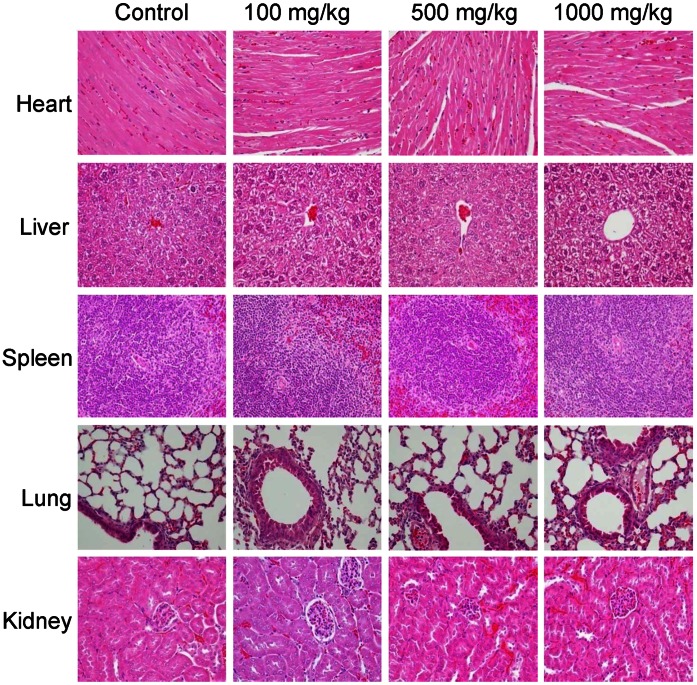
Effect of WCHF on the histopathological parameters of vital organs of test mice in subacute toxicity test. ICR mice received daily oral administration of normal drinking water or WCHF (100, 500 and 1000 mg/kg body weight) for 28 days. Representative H&E-stained tissue sections of heart, liver, spleen, lung and kidney are showed at 400× magnification.

Although *W. chinensis* has a long history of use as a medicinal or dietary herb [15], to the best of our knowledge, an evidence-based toxicity study of this plant has not been previously reported. To evaluate possible toxicity of *W. chinensis*, we performed a 28-day subacute oral toxicity test to determine the effect of different doses of WCHF (100, 500, and 1000 mg/kg body weight) in ICR mice. All treated mice exhibited normal morphological and behavioral phenotypes and growth rates, and survived well throughout the entire experimental period. No significant changes were observed in food intake activities, or in initial and final body weight among the four test groups (Table 1). Macroscopic observations of all organs excised from sacrificed mice were also found to be normal. In addition, there were no significant changes in the relative weight of vital organs in any of the tested groups (Table 1). At the tissue level, histological examination of vital organs showed no apparent tissue damage as a result of WCHF treatment (Fig. 4). Liver tissue sections showed a slightly diffused pattern of glycogen infiltration with vacuolization, which we suspect might have been caused by the non-fasting measure taken before sacrificing test mice. As compared with the control group, WCHF treatment did not result in a significant change for most of the hematological or blood biochemical parameters in test mice (see Table S2 in File S1), except for alanine aminotranserase (ALT) and aspartate aminotransferase (AST), which showed a detectable increase at the mid and high dosages (500 and 1000 mg/kg body weight) tested. These dosage levels are 10 and 20 times higher than the working dose (50 mg/kg body weight) we employed for this study. These results thus suggest that routine, daily oral administration of WCHF extract at dosages of 50 or even up to as high as 100 mg/kg body weight can be considered safe for test mice.

**Table 1:** Effect of WCHF on body weight, food intake and relative organ weight of test mice in subacute toxicity study.

			WCHF^a^ (mg/kg BW^b^)	
Group	Control	100	500	1000
Initial BW (g)	25.25±3.93	22.53±2.34	24.00±2.39	23.74±1.51
Final BW (g)	33.35±4.36	32.76±2.06	34.38±1.83	31.11±2.87
Food intake (g/mice/day)	4.68±0.90	4.39±0.93	4.64±1.07	4.58±1.50
Wet Organ/body weight ratio (%)				
Heart	0.63±0.09	0.62±0.06	0.63±0.06	0.64±0.09
Liver	6.50±0.22	6.24±0.51	6.38±0.04	6.63±1.16
Spleen	0.33±0.04	0.36±0.02	0.34±0.07	0.42±0.12
Lung	0.67±0.02	0.70±0.07	0.72±0.08	0.80±0.16
Kidney	0.88±0.03	0.93±0.08	0.95±0.06	0.89±0.14

Data are expressed as mean ± SD. (n = 6).

a WCHF = hot water extract of fresh *W. chinensis*.

b BW = body weight. There were no statistical differences between control and test groups.

## Discussion

In recent years the use of herbal remedies or medicinal foods has become a recognized strategy to combat human diseases such as IBD [10], [11]. *W. chinensis* is a traditional medicinal herb whose leaf and stem tissues are very commonly used as dietary supplements or health care products in many Asian countries. For example, *W. chinensis* is a key ingredient of a spectrum of commercial herbal teas currently sold in supermarkets in Taiwan. In our previous *in* *vitro* study, we established that *W. chinensis* extracts could significantly decrease lipopolysaccharide (LPS)-induced, transgenic nuclear factor- kappa B (NF-κB) and tumor necrosis factor-alpha (TNF-α) promoter activities in a mouse macrophage cell line, RAW 264.7 cells (data not shown). It also inhibited the expression of pro-inflammatory mediators, including the NF-κB, iNOS and cyclooxygenase-2 in LPS-stimulated cells. In light of these findings on the anti-inflammatory effect of *W. chinensis,* in this study we investigated the *in* *vivo* effect of *W. chinensis* on a DSS-induced murine colitis model.

The DSS-induced colitis mouse model mimics a number of symptoms and histopathological characteristics of IBD found in humans, including diarrhea, rectal bleeding, extensive crypt destruction, infiltration with granulocytes, edema and ulceration in colon tissue [18], [19], [33]. Using this model, we found that *W. chinensis* could effectively attenuate specific clinical symptoms and provide protection against the histopathological changes caused by colonic inflammation. Previous studies have shown that specific medicinal herbs, such as *Arctium lappa* (burdock) and *Scutelleria baicalensis*, can confer anti-colitis activities [34], [35]. Comparison with results from these studies suggested that WCHF extract from *W. chinensis* had a considerably lower effective dosage (e.g., 50, 100 and 1000 mg/kg for *W. chinensis*, *A. lappa* and *S. baicalensis*, respectively) than the previously tested medicinal plants [34], [35].

It is well recognized that specific tissue extractions, preparations and fractionation methods can affect the medicinal or nutritional properties of final extracts of medicinal or dietary plant materials [36]. For example, Capecka et al. [37] found significant differences in the antioxidant activities of fresh versus dry preparations of some *Lamiaceae* species. To investigate the optimal method for extraction of *W. chinensis* plant tissues with high anti-colitis efficacy, we adopted various methods commonly used by practitioners of traditional Chinese medicine (TCM) to prepare whole plant extracts, such as decoction of fresh or shade-dried *W. chinensis* plants in boiling water (WCHF and WCHD), or prolonged imbibitions of fresh *W. chinensis* plants in different concentrations of alcoholic solvent, e.g., in ethanol (as in WC50 and WC100). Interestingly, WCHF was found to be the most effective at protecting against DSS-induced colitis (Fig. 2). One possible explanation for this variation in efficacy might be the presence of specific polar compounds in fresh *W. chinensis* tissues that can only be effectively extracted by highly polar solvents such as water. Another possible explanation may be the increase in solubility in certain active phytochemicals that exist in WCHF due to boiling and associated physical/chemical activities. It has been reported that boiling in water is able to break the cellular constituents and increase the solubility of phenols in test plant tissues [38], [39]. Based on these findings, we suggest that boiling water extraction of plant tissue may provide a convenient and hygienic approach for family food preparation and industrial processing of anti-colitis natural products using *W. chinensis* as the starting or basic material.

With regard to the possible mode of action, immunohistochemical analysis showed that varying levels of inhibition of MMP-9 and iNOS expression were detected in colon tissues of test mice treated with different *W. chinensis* extracts (Fig. 2D, 2E). These results are in agreement with the data obtained from the anti-colitis activity in test mice, suggesting that the anti-colitis activity of *W. chinensis* may be associated with the inhibition of MMP-9 and iNOS expression. Upregulation of MMP-9 is known to be closely associated with IBD [40], [41], although the role of iNOS in IBD remains controversial [42]. Recently, Kretzmann et al. [43] showed that inhibition of iNOS may contribute to the therapeutic effect of glutamine in rats with 2,4,6-trinitrobenzene sulfonic acid-induced colitis. Dijkstra et al. [44] also reported that the expression of epithelial iNOS is highly bacterium-specific and can correlate with the severity of the disease, suggesting an important role for this enzyme in the development of IBD. Therefore, the anti-colitis activity of *W. chinesis* may be mediated in part via its effect on inhibition of iNOS activation in colon tissues.

Recently, there has been renewed interest by scientists from diverse disciplines in the evaluation of single compounds derived from natural plants for candidate drug development [45], [46]. Metabolite profiling (chemical fingerprinting) showed two specific peaks in the HPLC chromatogram of WCHF that were not present in comparable profiling of the WCHD extract. These two peaks may represent the major active ingredients responsible for the observed anti-colitis activities present in the fresh, but not air-dried, plant tissues. The compositional differences between these two *W. chinensis* extracts may be associated with certain enzymatic activities that actively degrade or modify the specific plant metabolites during the process of air-drying fresh plant tissues. Moreover, we compared the HPLC profile of WCHF to several known phyochemicals from Compositae. However, these two peaks were not identified.

Safety is a primary concern when considering the use of specific herbs as plant-derived nutritional, medicinal or health-care products. The liver tissue sections of mice treated with WCHF showed a slight to moderately diffused pattern of swelling and vacuolization. On the other hand, since the control group mice also exhibited a similar level of vacuolization in the liver, we suggest this alteration might have been caused by the slight glycogen accumulation/infiltration activity resulting from the action of not fasting the mice before sacrifice [47], [48]. Blood biochemical parameters showed that two enzymes that have been employed as indicators of liver function, namely ALT and AST, were increased in test mice consuming a dose of 500 or 1000 mg/kg body weight of WCHF (Table S2 in File S1). The working dosage of our *W. chinensis* extracts that exhibited effective anti-colitis activity in mice was 50 mg/kg. [49] According to the conversion of animal doses to human equivalent dose (HED) based on body surface area (BSA), this dose is equivalent to 14.28 g of fresh, vegetative plant tissues per 70 kg body weight in the human system. Therefore, in order to cause elevated ALT and AST activities, in theory, a human subject would need to ingest about 142–284 g of fresh plant tissue, a dose of *W. chinensis* extract 10 to 20 times higher per kilogram of body weight than that found effective in mice in this study. In addition, *W. chinensis* has been reported to provide a protective effect on hepatotoxin-induced acute hepatotoxicity, and the effective dosage was reported to be 300 mg/kg mouse body weight [13]. These finding suggest a detailed and systematic analysis of the different plant extracts of *W. chinensis* is required to clarify the effect of high dosage on liver function. Based on the data from this subacute toxicity study, we suggest that daily oral administration of WCHF at the working dosage of 14.28 g per 70 kg body weight would not result in any adverse health effects in humans.

In conclusion, this study shows that *W. chinensis* is non-toxic, and can actively ameliorate DSS-induced acute colitis in mice. Among different methods of preparation, extraction of *W. chinensis* with boiling water had the highest anti-colitis efficacy. This distinction in preparation method correlated with the difference in two specific peaks in the metabolite profiles of the WCHF and WCHD extracts. HPLC data suggest that specific active compounds present as minor but detectable metabolite profile peaks may confer the anti-inflammatory effect of *W. chinensis* revealed in the DSS-induced colitis mouse model. Hot water extract of *W. chinensis* may be a promising phyto-therapeutic agent for IBD, and perhaps other diseases characterized by intestinal inflammation. In the future, further studies to identify the possible synergistic properties of the active phytocompound(s) responsible for the anti-colitis activity of *W. chinensis* are needed. In addition, it will also be important to consider the applications of crude extract of *W. chinensis,* which contains a cocktail of different phytochemicals, as therapeutic agent against colitis. As only one species of the *Wedelia* genus was used in our study, it will further be important to evaluate the potential anti-inflammatory activities of other plant cultivations or species of the *Wedelia* genus for use as evidence-based nutraceuticals, health care agents or nutritional supplements. And since *W. chinensis* is a fast growing plant, and it is crispy and succulent in taste, we also suggest, based on food science definition, that this plant could be promoted into a new vegetable plant for nutritional and dietary use.

## Supporting Information

File S1 **Contains Table S1 and Table S2.** Table S1, Scoring system for histological pathology study. Table S2, Effect of WCHF on hematological parameters (A) and blood biochemical parameters (B) of test mice in subacute toxicity study.
(DOC)
