# Supplementary material for: Dietary Uptake of Wedelia chinensis Extract Attenuates Dextran Sulfate Sodium-Induced Colitis in Mice
Source: PLoS One. 2013 May 29;8(5):e64152. doi: 10.1371/journal.pone.0064152 (PMC3667021; doi:10.1371/journal.pone.0064152)
Supplement: File S1 — Contains Table S1 and Table S2. Table S1, Scoring system for histological pathology study. Table S2, Effect of WCHF on hematological parameters (A) and blood biochemical parameters (B) of test mice in subacute toxicity study. (DOC) [file pone.0064152.s001.doc]

**Table S1**

**Scoring system for histological pathology** study

| Organ/Histopathological finding | Histological score^a^ |
| --- | --- |
| Loss, crypt | 1-5 |
| Regeneration, crypt | 1-5 |
| Edema, submucosa | 1-5 |
| Inflammation, mononuclear cells | 1-5 |
| Ulcer, with fibroblast cell infiltration | 1-5 |

Adopted from Cooper et al. [19].  ^a^Degree of lesions is graded from one to five depending on severity: 1 = minimal (< 1%); 2 = slight (1-25%); 3 = moderate (26-50%); 4 = moderate/severe (51-75%); 5 = severe/high (76-100%).

**Table S2**

**Effect of WCHF on hematological parameters (A) and blood biochemical parameters (B) of test mice in subacute toxicity study**

(A) Hematological parameters

|  |  | WCHF (mg/kg BW) | | | |
| --- | --- | --- | --- | --- | --- |
| Parameter (unit) | Control | | 100 | 500 | 1000 |
| RBC (×10^12^/l) | 9.11 ± 0.56 | | 9.32 ± 0.27 | 9.37 ± 0.25 | 9.65 ± 0.48 |
| Hemoglobin (g/dl) | 15.7 ± 1.0 | | 15.2 ± 0.5 | 15.6 ± 0.2 | 16.1 ± 0.2 |
| Hematocrit (%) | 52.5 ±3.4 | | 51.2 ± 0.7 | 52.0 ± 0.8 | 53.0 ± 0.7 |
| MCV (fl) | 57.6 ± 1.3 | | 55.0 ± 1.3 | 55.6 ± 1.9 | 56.0 ± 2.4 |
| MCH (pg) | 17.2 ± 0.3 | | 16.3 ± 0.4 | 16.6 ± 0.6 | 21.3 ± 9.1 |
| MCHC (g/dl) | 30.0 ± 0.1 | | 29.7 ± 0.5 | 29.9 ± 0.3 | 30.3 ± 0.3 |
| Platelet (×10^9^/l) | 715.5 ± 283.9 | | 881.0 ± 236.9 | 879.8 ± 311.4 | 823.8 ± 291.9 |
| WBC (×10^9^/l) | 7.53 ± 1.56 | | 7.68 ± 1.50 | 7.85 ± 1.63 | 9.27 ± 3.94 |
| NEU (%) | 19.6 ± 9.1 | | 20.9 ± 8.8 | 22.2 ± 7.0 | 22.7 ± 12.8 |
| LYM (%) | 71.2 ± 9.0 | | 72.0 ± 7.0 | 68.9 ± 8.9 | 64.9 ± 19.6 |
| MONO (%) | 2.43 ± 0.43 | | 1.84 ± 0.24 | 2.76 ± 0.88 | 2.33 ± 0.41 |
| EOS (%) | 3.38 ± 0.84 | | 3.19 ± 1.03 | 3.39 ± 0.97 | 6.74 ± 7.84 |
| BASO (%) | 3.43 ± 0.38 | | 2.57 ± 0.81 | 3.20 ± 0.81 | 3.33 ± 1.00 |

(B) Blood biochemical parameters

|  |  | WCHF (mg/kg BW) | | |
| --- | --- | --- | --- | --- |
| Parameter (unit) | Control | 100 | 500 | 1000 |
| Total proteins (g/dl) | 5.4 ± 0.2 | 5.6 ± 0.4 | 5.4 ± 0.2 | 5.5 ± 0.2 |
| Albumin (g/dl) | 2.90 ± 0.08 | 3.00 ± 0.29 | 2.95 ± 0.06 | 3.10 ± 0.26 |
| BUN (mg/dl) | 27.6 ± 3.3 | 25.8 ± 2.4 | 22.8 ± 1.7^*^ | 23.8 ± 4.0 |
| Creatinine (mg/dl) | 0.23 ± 0.05 | 0.18 ± 0.05 | 0.25 ± 0.06 | 0.20 ± 0.07 |
| Calcium (mg/dl) | 9.98 ± 0.22 | 10.48 ± 0.42 | 10.10 ± 0.32 | 10.20 ± 0.54 |
| Phosphorus (mg/dl) | 10.35 ± 0.71 | 9.73 ± 0.92 | 10.38 ± 1.14 | 10.30 ± 0.85 |
| Glucose (mg/dl) | 166.5 ± 26.7 | 155.8 ± 24.5 | 151.3 ± 6.6 | 153.3 ± 40.8 |
| AST (U/l) | 102.5 ± 24.2 | 126.3 ± 34.8 | 136.8 ± 12.2^*^ | 366.5 ± 184.3^*^ |
| ALT (U/l) | 74.5 ± 18.0 | 67.3 ± 19.3 | 158.0 ± 61.7^*^ | 170.0 ± 78.7^*^ |
| ALP (U/l) | 298.0 ± 38.0 | 308.0 ± 52.9 | 282.5 ± 32.6 | 273.0 ± 144.7 |
| Bilirubin (mg/dl) | 0.4 ± 0.2 | 0.3 ± 0.2 | 0.3 ± 0.1 | 0.3 ± 0.1 |

Data are expressed as mean ± SD. MCV = mean corpuscular volume, MCH = mean corpuscular hemoglobin, MCHC = mean corpuscular hemoglobin concentration, RDW = red cell distribution width, MPV = mean platelet volume, PCT = plateletcrit, PDW = platelet distribution width, NEU = neutrophil count, LYM = lymphocyte count, MONO = monocyte count, EOS = eosinophil count, BASO = basophil count. AST = aspartate aminotransferase, ALT = alanine aminotransferase, ALP = alkaline phosphatase, TB = total bilirubin, BUN = blood urea nitrogen. There were no significant differences between control and test groups.
